# Varicella-zoster virus glycoprotein expression differentially induces the unfolded protein response in infected cells

**DOI:** 10.3389/fmicb.2014.00322

**Published:** 2014-07-01

**Authors:** John E. Carpenter, Charles Grose

**Affiliations:** Virology Laboratory, Department of Infectious Diseases, University of Iowa Children's HospitalIowa City, IA, USA

**Keywords:** herpesvirus, unfolded protein response, autophagy, tunicamycin, ERAD, CREBH, gp78, INSIG

## Abstract

Varicella-zoster virus (VZV) is a human herpesvirus that spreads to children as varicella or chicken pox. The virus then establishes latency in the nervous system and re-emerges, typically decades later, as zoster or shingles. We have reported previously that VZV induces autophagy in infected cells as well as exhibiting evidence of the Unfolded Protein Response (UPR): XBP1 splicing, a greatly expanded Endoplasmic Reticulum (ER) and CHOP expression. Herein we report the results of a UPR specific PCR array that measures the levels of mRNA of 84 different components of the UPR in VZV infected cells as compared to tunicamycin treated cells as a positive control and uninfected, untreated cells as a negative control. Tunicamycin is a mixture of chemicals that inhibits N-linked glycosylation in the ER with resultant protein misfolding and the UPR. We found that VZV differentially induces the UPR when compared to tunicamycin treatment. For example, tunicamycin treatment moderately increased (8-fold) roughly half of the array elements while downregulating only three (one ERAD and two FOLD components). VZV infection on the other hand upregulated 33 components including a little described stress sensor *CREB-H* (64-fold) as well as ER membrane components *INSIG* and *gp78*, which modulate cholesterol synthesis while downregulating over 20 components mostly associated with ERAD and FOLD. We hypothesize that this expression pattern is associated with an expanding ER with downregulation of active degradation by ERAD and apoptosis as the cell attempts to handle abundant viral glycoprotein synthesis.

## Introduction

VZV is a human pathogen that spreads to children as varicella or chicken pox and re-emerges later as zoster or shingles (Ross, 1962; Grose, 1981). VZV is one of nine human herpesviruses (Davison, 2010). The virus is supremely adapted to its human host and infects most people in a given community (Hope-Simpson, 1965; Choo et al., 1995). It is endemic throughout the world but largely controlled in some countries by vaccination with a live attenuated virus (Seward et al., 2008; Marin et al., 2011).

Varicella infection, within its natural human host, spreads from the nasopharynx via infection of a limited number of T cells that home to the skin epidermis (Arvin et al., 2010). Once there the infection is passed to the basal keratinocytes making up the innermost layer of the epidermis (Ku et al., 2004). The virus progressively infects other cells in its proximity until reaching the surface of the skin in the form of characteristic VZV vesicles. Within the area of the vesicle, polykaryocytes or multi-nucleated cells are found due to VZV-induced cell to cell fusion (Weigle and Grose, 1984). As the number of viral particles increase within the vesicle, some particles travel retrograde along sensory neurons in the skin to the sensory ganglia emanating from the spinal cord (Gilden et al., 2003). In the ganglia, the virus becomes latent or quiescent until much later (years or decades) in the life of the host. Under conditions of immunosuppression or aging, VZV can reactivate within the ganglia and spread back anterograde to the skin to cause zoster or shingles (Arvin, 1987). Typically, this event only happens from a single ganglion within one dermatome (Hope-Simpson, 1965).

VZV is an alphaherpesvirus that exists as a multilayered structure approximately 200 nm in diameter (Grose et al., 1983). In the virus particle, the genome (dsDNA) is surrounded by a protein capsid structure that is covered by an amorphous layer of tegument proteins. These two structures are surrounded by a lipid envelope that contains viral glycoproteins. The VZV genome is the smallest of the human herpesviruses and encodes at least 71 unique proteins (ORF0–ORF68) with three more opening reading frames (ORF69–ORF71) that duplicate earlier open reading frames (ORF64–62, respectively) (Davison and Scott, 1986). Only a fraction of the encoded proteins form the structure of the virus particle (Kinchington et al., 1992). Among those proteins are nine glycoproteins: ORF5 (gK), ORF9A (gN), ORF14 (gC), ORF31 (gB), ORF37 (gH), ORF50 (gM), ORF60 (gL), ORF67 (gI), and ORF68 (gE). Abundant biosynthesis of viral glycoproteins increases to the point of excluding cellular glycoprotein expression under conditions of infection in cultured cells (Grose, 1980).

Of importance, VZV induces autophagy in infected cells as well as exhibiting evidence of the Unfolded Protein Response (UPR): XBP1 splicing and a greatly expanded ER (Takahashi et al., 2009; Carpenter et al., 2011). More recently, we found that inhibition of autophagy by either 3-methyl adenine (3-MA) treatment or siRNA knockdown of ATG-5, a necessary autophagy protein, reduced glycoprotein expression and altered post-translational modifications of VZV gE and gI and ultimately VZV infectivity in culture. (Buckingham et al., 2014). These results highlight the role of VZV glycoprotein expression in inducing ER stress and associated autophagy. Our observations of enlarged ER and spliced XBP-1 in VZV infected cells led us to consider what other elements of the UPR are being activated. We decided to use a commercial PCR array that measures the levels of transcripts of 84 different components of the UPR. Herein we report the results of comparing VZV infected cells vs. tunicamycin treated cells with this UPR PCR array.

## Methods

### Viruses and cells

VZV-32 is a low passage laboratory strain; its genome has been completely sequenced and falls within European clade 1 of VZV genotypes (Peters et al., 2006). MRC-5 human fibroblast cells or HeLa cells were grown in six well tissue culture plates with and without 12 mm round or 22 mm square coverslips in Minimum Essential Medium (MEM; Gibco, Life Technologies) supplemented with 7% fetal bovine serum (FBS), L-glutamine, non-essential amino acids, and penicillin/streptomycin. When monolayers were nearly confluent, MRC-5 cells were inoculated with VZV-infected cells at a ratio of one infected cell to eight uninfected cells by previously described methods (Grose and Brunel, 1978).

### Transfection

HeLa cells were transfected with plasmids containing VZV gE (pTargeT_gE) or VZV ORF62 (pCMV_IE62) under the CMV promoter as described previously (Carpenter et al., 2011). The plasmids were transfected into HeLa cells using ExtremeGene HP (Roche) transfection reagent (Jacobsen et al., 2004) at 10 μl/ml and plasmid DNA at a concentration of 1.0 μg/ml. After 6 h, the culture medium was replaced with plasmid/transfection reagent free medium. At 24 h post-transfection, RNA was extracted from all wells in a culture plate and cells incubated on coverslips were fixed and processed for microscopy.

### Real-time RT-PCR

Total RNA was extracted from uninfected, tunicamycin treated and VZV infected fibroblast cells in six well plates at the given time points using the RNEasy mini kit (Qiagen). RNA quality and quantity was assayed by UV spectroscopy using a NanoDrop spectrometer. Both A260/A280 and A260/A230 ratios were within 20% of 2.0 and infected cells from a six well plate well (6.5 sq cm) yielded approximately 3 μg of RNA in 60 μl. Further, the RNA was electrophoresed in an Agilent Bioanalyzer 2100 (Agilent) and yielded RIN values within 20% of 10. Polyadenylated RNA was converted to cDNA using anchored Oligo(dT) primers and the SuperScript III First-Strand Synthesis System for RT-PCR (Invitrogen) to yield approximately 20 ng of cDNA. The entire cDNA sample from one well of cells was mixed into 1 ml of 1× diluted Power SYBR Green Master Mix (ABI) and split into all wells of a SA Biosciences UPR PCR array (Life Technologies) with a multichannel pipettor (25 μl per well). The measurements were carried out in triplicate using cDNA from three of the original six wells in the plate for all types of samples using a Model 7000 real time PCR instrument (ABI). The resulting PCR results were processed using the SDS 1.2.3 software (Applied Biosystems). C_T_ values of each measurement were normalized to an average of 16.0 for housekeeping genes (wells H1–H5 of the UPR array) to form ΔC_T_ values which were then used to calculate averages and standard deviations between triplicate measurements. Subsequent ΔΔC_T_ values were calculated by differences between averages of VZV infected ΔC_T_ values or tunicamycin treated ΔC_T_ values with the average uninfected ΔC_T_ values. Uncertainties correspond to propagation of errors using standard deviations between the uninfected and infected or tunicamycin averages.

### RT-PCR primers

To confirm measurements from the UPR specific PCR array, several RT-PCR measurements were carried out using the following primers: BiP: forward 5′-CCC CAA CTG GTG AAG AGG AT-3′ and reverse 5′-GCA GTA AAC AGC CGC TTA GG-3′; DNAJB9/ERDj4: forward 5′-ACA TCT GTG ACT TGC GTT GC-3′ and reverse 5′-TGG GCA ATA AAA CCA TTT CC-3′; CREBH: forward 5′-GGG AGA CGA GCT GTG AGC-3′ and reverse 5′-TGT CTG AGT GTC GGT TCC TG-3′; PERK: forward 5′-GCC TAA GGA GGT AGC AGC AA-3′ and reverse 5′-GGG ACA AAA ATG GAG TCA GC-3′.

### Antibodies

Murine MAb antibodies to VZV gE (3B3) and IE62 (5C6) produced in our laboratory were used in addition to a rabbit polyclonal antibody to LC3B (Santa Cruz Biotech sc-28266).

### Imaging protocols

Samples of infected and uninfected cells were prepared for confocal microscopy by methods described previously (Carpenter et al., 2008). Briefly, the samples were fixed with paraformaldehyde and permabilized with 0.05% Triton-X-100 in PBS and then blocked in 5% non-fat milk with 2.5% normal goat serum for 2 h at RT. The primary antibody (1:2000) was added for 2 h at RT and overnight at 4°C. After washing (3 × 5 min with PBS) the samples were incubated with the secondary antibody (1:1250) and the Hoechst 33342 dsDNA stain (1:500) for 2 h at RT then washed before mounting on slides for viewing. Following preparation, the samples were viewed on a Zeiss 710 confocal fluorescent microscope (Duus et al., 1995).

### Tunicamycin protocol

Conditions for treatment of cultured cells with tunicamycin (2.5 μg/ml; Calbiochem, #654380) have been described in earlier papers in which we were investigating VZV glycoprotein biosynthesis (Montalvo et al., 1985; Carpenter et al., 2010). For experiments in uninfected cells, tunicamycin (2.5 μg/ml) was added 24 h after subculturing and the monolayer was fixed after another 24 h.

### ER labeling by dicarbocyanine dye

DiOC_6_ (3-3-dihexyloxa-carbocyanine iodide) was obtained in powder form from Molecular Probes (D-273) and dissolved (0.7 mg/ml) in ethanol (Sabnis et al., 1997). An aliquot of the DiOC_6_ stock (2.8 μl/ml yielded a final concentration of DiOC_6_ of 2 μg/ml) was added to warm cell culture medium; this medium was applied to live cells for 30 min, then rinsed 2× with PBS and processed for fluorescent microscopy as described above.

## Results

### VZV infected cells exhibited abundant glycoprotein expression with an enlarged ER and increased autophagy

Within cell culture, VZV is entirely cell associated with no release of cell-free virus (Grose and Brunel, 1978; Weller, 1983). Monolayers are inoculated with VZV-infected cells. The susceptibly of cells to VZV determines how long it takes to infect the whole monolayer but spread typically requires 3–5 days. Within infected cell monolayers, we observe a range of fused cells. For example, VZV induces massive syncytia involving hundreds of nuclei in melanoma cells while VZV infection of less fusogenic cells such as lung fibroblasts or skin keratinocytes induces syncytia involving tens of nuclei.

**Figure 1:**
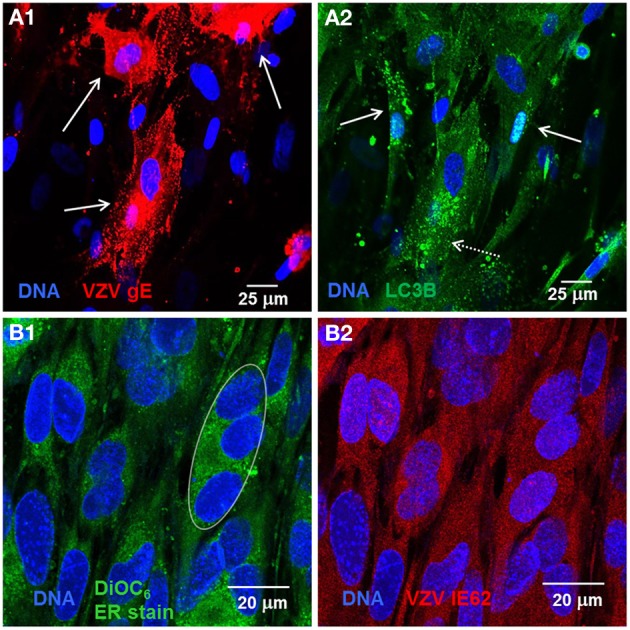
**VZV infected cells exhibited abundant glycoprotein expression, increased autophagosomes, and an enlarged ER**. Human fibroblast cells (MRC-5) were grown on glass coverslips in tissue culture plates until 60% confluent and then infected with VZV-32 infected MRC-5 cells at a ratio of 1:8. At 48 hpi, the cultures were fixed, permeabilized, blocked and immunolabeled for VZV gE, IE62, or cellular LC3B. The cells were then imaged at 400× with a Zeiss 710 confocal fluorescence microscope. Other cultures grown and infected similarly were incubated in medium containing DiOC6, a polar dye, prior to fixation. **(A1)** VZV glycoprotein gE expression is abundant in three cells (solid arrows) in the image. **(A2)** LC3 puncta indicative of autophagosomes are apparent in cells expressing gE (arrows, dashed line) and nearby cells that are newly infected (arrows, solid line). **(B1)** DiOC_6_ staining in 19 cells at 630×. A representative white ellipse indicates the enlarged ER in an infected syncytium. **(B2)** VZV IE62 staining indicates that all 19 cells are infected and several are in syncytia.

Recently, we observed that VZV induces increased LC3-positive puncta formation indicative of autophagosomes within cultured cells as well as from cells removed from varicella and zoster vesicles (e.g., Figures 1A1,A2) (Takahashi et al., 2009). Unlike the closely related herpes simplex virus, VZV does not encode any known inhibitors of autophagy, such as ICP 34.5. Later we observed that VZV infected cells also exhibited signs of ER stress, namely *XBP-1* splicing and a greatly enlarged ER (see, e.g., Figures 1B1,B2). The latter results led to the hypothesis that VZV glycoprotein synthesis induces ER stress that is partially relieved by an enlarging ER and increased autophagy (Carpenter et al., 2011).

### UPR gene transcription was different in VZV infected cells vs. tunicamycin treated cells

Based on the observations in the previous section, we sought to further document the induction of the UPR within VZV infected cells via a UPR-specific PCR array manufactured by SA Biosciences (now part of Qiagen). This 96 well plate consists of 84 wells containing primers to the 3′ Untranslated Region (UTR) of transcripts associated with the UPR and the remaining 12 wells containing primers to housekeeping genes and PCR and cDNA quality control wells. Table 1 lists the UPR specific primers or wells where the wells are grouped by association with a given UPR function: ANTI or PRO (anti or pro-apoptotic), ERAD (ER associated degradation), FOLD (primarily folding chaperones), LIPID (transcripts associated with lipid synthesis and metabolism), SENSOR (transcripts associated with ER membrane resident proteins known to “sense” and signal ER stress conditions), TF (other transcription factors like C/EBPβ) and finally TRANS for two components associated with protein translation. Each group will be described more fully in the next sections.

**Table 1:** **UPR qPCR results for tunicamycin treated and VZV infected cells**.

**Gene**	**Function**	**Group**	**TM treated**		**VZV infected**	
			**ΔΔC_T_**	**STD**	**ΔΔC_T_**	**STD**
ARMET/MANF	ERSE-II regulated; reduces cell proliferation and UPR initiated apoptosis	ANTI	5.0	0.2	6.1	0.5
EDEM3	ER degradation enhancer, mannosidase alpha-like 3	ERAD	−1.5	0.9	−4.7	0.9
PPIA	Peptidylpropyl isomerase A (cyclophilin A)	ERAD	−1.4	0.2	−0.7	0.5
UBE2G2	E2 ubiquitin conjugating enzyme G2	ERAD	−0.9	0.2	0.4	0.5
NPLOC4 (NPL4)	Regulates poly Ub on cytosolic side of ER membrane with VCP	ERAD	−0.7	0.2	1.9	0.5
UBXN4/erasin	UBX domain protein 4—adaptor protein to VCP	ERAD	−0.5	0.3	−6.1	0.5
USP14	Ubiquitin specific peptidase 14 in cytosol	ERAD	−0.4	0.3	−0.3	0.5
SEC62	ERAD translocation pore formation	ERAD	−0.2	0.3	−4.3	0.4
UFD1L	Regulates poly Ub on cytosolic side of ER membrane with VCP	ERAD	0.1	0.1	0.9	0.4
UBE2J2	E2 ubiquitin conjugating enzyme J2	ERAD	0.2	0.1	−0.4	0.4
ATXN3	Ataxin 3—deubiquiting enzyme	ERAD	0.8	0.2	3.8	0.5
FBX06	E3 ubiquition ligase of glycoproteins in ER lumen	ERAD	0.9	0.1	−2.1	0.4
RNF5	Ring Finger protein 5—E3 Ubiquitin ligase in ER membrane	ERAD	1.1	0.1	1.1	0.4
DERL1	Derlin family member E3 Ubiquitin ligase in ER membrane	ERAD	1.2	0.2	2.0	0.5
VCP(p97)	Regulates poly Ub of translocated ER substrates with NPL4 and UFD1L	ERAD	1.3	0.2	−0.4	0.5
EDEM1	ER degradation enhancer, mannosidase alpha-like 1—trims mannose	ERAD	1.4	0.2	1.5	0.4
SEL1L	Adaptor protein of Derlin-3/HRD1 in ER membrane	ERAD	2.0	0.3	−2.6	0.4
OS9	Glycoprotein protein quality control	ERAD	2.0	0.2	−0.7	0.5
DERL2	Derlin family member, E3 Ubiquitin ligase in ER membrane	ERAD	2.4	0.2	−1.3	0.5
SYVN1 (DER3/HRD1)	Synoviolin, Derlin family member E3 Ubiquitin ligase in ER membrane	ERAD	2.7	0.1	1.9	0.4
SELS	Selenoprotein S—oxidoreductase (oxidative stress)	ERAD	3.0	0.2	1.6	0.5
HERPUD1 (HERP)	Mediates degradation of ER Ca channels	ERAD	4.3	0.3	−1.9	0.5
HSPA2	HSP70 protein 2	FOLD	−1.5	0.3	1.2	0.6
HSPA4	HSP70 protein 4	FOLD	−0.8	0.3	1.4	0.5
HSPA1B	HSP70 protein 1B	FOLD	−0.5	0.2	0.0	0.5
GANAB (Glu II)	Glucosidase that trims N-linked glycans	FOLD	−0.5	0.3	−1.6	0.5
PRKCSH	Protein kinase C substrate 80K-H (subunit of glucosidase II)	FOLD	−0.4	0.2	1.7	0.5
GANC	Glycosal hydoloysis	FOLD	−0.2	0.2	2.3	0.4
TCP1	Component of Chaperonin	FOLD	−0.2	0.1	−0.7	0.4
HSPA1L	HSP70 protein 1 like	FOLD	−0.1	0.2	−3.0	0.4
HSPA4L	HSP70 protein 4 like	FOLD	0.0	0.3	−1.7	0.5
CCT4	Chaperonin containing TCP1, subunit 4 (delta)	FOLD	0.3	0.1	−1.3	0.4
CCT7	Chaperonin containing TCP1, subunit 7 (eta)	FOLD	0.4	0.2	0.4	0.5
HSPH1	HSP105 protein 1	FOLD	0.6	0.1	1.6	0.4
PFDN5	Prefoldin subunit 5; co-chaperone of Chaperonin complex	FOLD	0.7	0.2	2.2	0.5
PFDN2	Prefoldin subunit 2	FOLD	1.0	0.2	1.5	0.5
TOR1A	Torsion A—ATPase	FOLD	1.0	0.2	−1.0	0.5
UGCGL2 (UGT2)	UDP-glucose ceramide glucosyltransferase-like 2	FOLD	1.0	0.2	1.6	0.5
UGCGL1 (UGT1)	UDP-glucose ceramide glucosyltransferase-like 1	FOLD	1.1	0.2	0.9	0.5
ERP44	Thiol chaperone	FOLD	1.5	0.3	−1.8	0.5
CALR	Calreticulin; glycoprotein folding chaperone	FOLD	1.5	0.3	−1.1	0.5
RPN1	Ribophorin 1—substrate specific facilitator of N-glycosylation	FOLD	1.6	0.2	−1.6	0.5
ERO1L	Thiol oxidase governs redox state of ER (with Ca2+)	FOLD	1.6	0.3	0.8	0.5
DNAJC10 (ERdj5)	DNAJ (HSP40 homolog), subfamily C, member 10	FOLD	1.7	0.3	−1.0	0.5
DNAJB2	DNAJ (HSP40 homolog), subfamily B, member 2	FOLD	1.8	0.2	2.0	0.5
SEC63	Regulates ER import of membrane proteins	FOLD	2.1	0.2	0.3	0.4
CANX	Calnexin; glycoprotein folding chaperone; binds Ca2+	FOLD	2.2	0.3	1.2	0.5
SIL1(BAP)	Nucleotide exchange factor; binds BiP	FOLD	2.4	0.1	2.0	0.4
PDIA3 (ERP57)	Protein disulfide isomerase family A, member 3	FOLD	2.5	0.3	1.9	0.5
DNAJC3	DNAJ (HSP40 homolog), subfamily C, member 3	FOLD	2.6	0.1	2.4	0.4
DNAJC4	DNAJ (HSP40 homolog), subfamily C, member 4	FOLD	2.9	0.2	2.9	0.5
ERO1LB	Thiol oxidase governs redox state of ER (with Ca2+)	FOLD	3.8	0.2	1.5	0.4
DNAJB9 (ERdj4)	DNAJ (HSP40 homolog), subfamily B, member 9	FOLD	5.5	0.2	−2.9	0.5
HSPA5	HSP70 protein 5 GRP78 (BIP)	FOLD	6.3	0.1	4.7	0.4
SREBF2	Sterol regulatory element binding TF 2	LIPID	0.9	0.1	−1.9	0.4
RNF139 (TRC8)	E3 Ubiquition ligase associated with INSIG	LIPID	−0.3	0.6	−1.2	0.6
INSIG2	Insulin induced protein isoform 2; regulation of cholesterol synthesis	LIPID	0.3	0.3	4.1	0.4
INSIG1	Insulin induced protein isoform 1; regulation of cholesterol synthesis	LIPID	0.5	0.3	5.3	0.4
AMFR (gp78)	Autocrine motility factor receptor; E3 Ub ligase; regulation of cholesterol	LIPID	0.6	0.3	6.2	0.5
SREBF1	Sterol regulatory element binding TF 1	LIPID	1.1	0.2	0.3	0.5
SCAP	Activates SREBF by cleaving it	LIPID	1.6	0.2	−0.3	0.5
SERP1 (RAMP4)	Stress induced ER protein 1; ER salt channel regulation	LIPID	2.7	0.3	2.9	0.6
MAPK8 (JNK1)	Map kinase K8 aka JNK1; pro-apoptotic in response to TNFα	PRO	−0.8	0.1	2.2	0.4
BAX	BCL2-associated X protein; induces release of COX-2 from mitochondria	PRO	0.5	0.3	−1.0	0.5
MAPK9 (JNK2)	Mitogen-activated protein kinase 9	PRO	0.5	0.2	1.0	0.4
HTRA2	HTRA serine peptidase 4	PRO	0.6	0.2	−0.2	0.5
MAPK10 (JNK3)	Map kinase K10 aka JNK3; pro-apoptotic in neurons	PRO	1.0	0.4	2.3	0.6
HTRA4	HTRA serine peptidase 2	PRO	1.4	0.4	4.0	0.6
CHOP	Aka DDIT3/GADD153; ER stress associated apoptotic protein	PRO	5.3	0.2	0.1	0.4
MBTPS2/S2P	Membrane bound TF peptidase, site 2 (active in Golgi)	SENSOR	−0.2	0.3	1.0	0.4
MBTPS1/S1P	Membrane bound TP peptidase, site 1 (active in Golgi; cleaves ATF6)	SENSOR	−0.1	0.2	0.1	0.4
ERN1 (IRE1α)	IRE1α is an endonuclease that splices XBP1 upon activation	SENSOR	0.0	0.2	1.6	0.4
ATF6B	ATF6 beta	SENSOR	0.5	0.3	−3.0	0.5
CREB3 (LUMAN)	OASIS (B-zip TF) family member; cell proliferation	SENSOR	1.4	0.2	1.0	0.4
ATF6	Activating transcription factor 6	SENSOR	1.5	0.2	0.3	0.5
EIF2AK3 (PERK)	ER stress sensor; PKR-like kinase	SENSOR	1.6	0.2	−4.2	0.5
ERN2 (IRE1β)	ER to nucleus signaling protein 2	SENSOR	1.8	0.5	3.1	0.6
NUCB1	Nucleobindin 1; negative regulation of ATF6	SENSOR	3.1	0.2	4.1	0.5
CREB3L3 (CREBH)	TF regulating lipogenesis and secretory pathway	SENSOR	4.4	1.6	9.3	0.9
XBP1	X box binding protein 1; splicing by IRE1 activates XBP1	TF	0.7	0.1	−0.4	0.4
CEBPB (C/EBPβ)	Bzip TF with wide impact on cell cycle and proliferation	TF	1.3	0.3	−2.9	0.6
ATF4	Activates stress response (including CHOP)	TF	1.7	0.2	1.5	0.5
EIF2A	Eukaryotic translation initiation factor 2A	TRANS	0.0	0.5	0.1	0.4
PPP1R15A	Protein phosphatase 1, subunit 15A	TRANS	1.8	0.2	1.1	0.4

Human fibroblast cells (MRC-5) were grown on glass coverslips in tissue culture plates then infected with VZV-32 infected MRC-5 cells or treated with tunicamycin (TM), a N-glycosylation inhibitor. At 72 hpi, RNA was extracted from the VZV-32 infected cultures. For the TM treated cultures, RNA was extracted at 24 h post-treatment. RNA from the VZV-32 infected, TM treated and uninfected cell cultures was then converted to cDNA which was applied to UPR specific PCR arrays (SA Biosciences) and real time PCR was carried out on an ABI 7000 PCR instrument The resulting CT values were then normalized (ΔC_T_) by housekeeping genes in the plate and then differences (ΔΔC_T_) between the uninfected and infected or tunicamycin treated values were computed and averaged. Abbreviations: anti-apoptotic (ANTI), ER associated degradation (ERAD), protein folding chaperones (FOLD), lipid and fat metabolism (LIPID), pro-apoptotic (PRO), ER stress sensor proteins (SENSOR), other transcription factors (TF) and protein translation associated proteins (TRANS). Error estimates correspond to standard deviation (STD).

**Figure 2:**
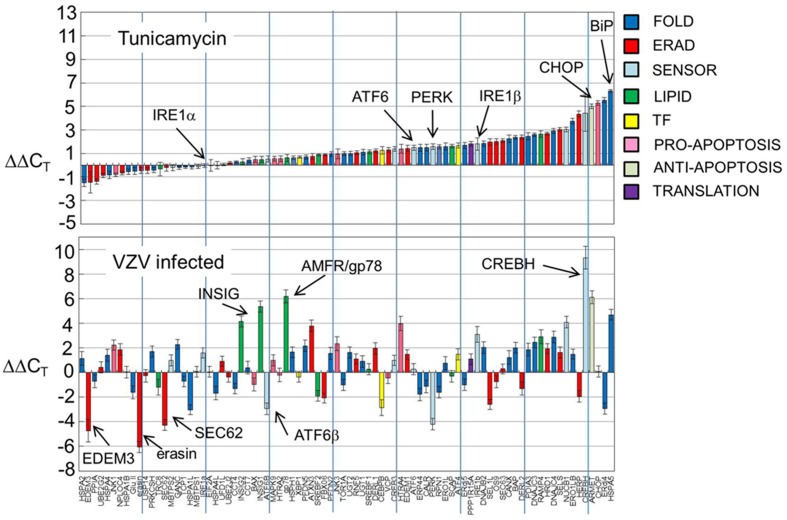
**UPR gene transcription was significantly different in VZV infected cells vs. either uninfected cells or tunicamycin treated cells**. Human fibroblast cells (MRC-5) were grown in tissue culture plates then infected with VZV-32 infected MRC-5 cells or treated with tunicamycin (TM), a N-glycosylation inhibitor. At 72 hpi, RNA was extracted from the VZV-32 infected cultures. For the TM treated cultures, RNA was extracted at 24 h post-treatment. RNA from the VZV-32 infected, TM treated and uninfected cell cultures was then converted to cDNA, which was applied to UPR specific PCR arrays (SA Biosciences); real time PCR was carried out on an ABI 7000 PCR instrument. The resulting C_T_ values were then normalized (ΔC_T_) by the housekeeping genes of the plate and differences (ΔΔC_T_) between the uninfected and infected or tunicamycin treated values were computed and averaged. Graphs of the resulting values show that tunicamycin treatment, a classical ER stressor, resulted in upregulation of 66 of the 84 UPR genes with known folding chaperones such as *BiP* (in blue). Also upregulated was the pro-apoptotic factor *CHOP*. By contrast, only 43 of the UPR genes were upregulated in VZV infected cells although several, such as *CREBH*, were more upregulated than in tunicamycin treated samples. Error bars correspond to standard deviation when averaging.

Gene transcripts were measured in uninfected human fibroblasts, tunicamycin (TM) treated fibroblasts and VZV infected fibroblasts. Each measurement was done in triplicate. The measured C_T_ values were normalized so that in each case the housekeeping gene transcripts measured C_T_ average was 16 and then the triplicate measurements were averaged and standard deviations computed to generate ΔC_T_. Differences between the uninfected ΔC_T_ and those associated with TM treated and VZV infected cell transcript measurements were then calculated to form the final measurements ΔΔC_T_ listed in Table 1. Graphs of the resulting values (Figure 2) showed that tunicamycin treatment, a classical ER stressor by inhibition of N glycosylation, upregulated 66 of the 84 UPR genes, with known folding chaperones, e.g., BiP (in blue), particularly upregulated. Also upregulated is the pro-apoptotic factor *CHOP* (pink). By contrast, only 43 of the UPR genes are upregulated in VZV infected cells. In particular, those genes most upregulated such as *CREB3L3/CREBH* (light blue) are more upregulated than after TM treatment. VZV infected cells also upregulated the LIPID transcripts *AMFR/gp78* and *INSIG* (green) while downregulating a number of ERAD components such as *UBXN4/erasin* and *EDEM3* (red). These differences will be considered by group in the subsequent sections.

### VZV infection significantly upregulated the transcription factor CREBH

The SENSOR grouping includes the best known ER stress sensors: *PERK, IRE1α* and *ATF6* but also two CREB proteins (*CREB3/LUMAN* and *CREBH*) as well as primers to the Golgi resident proteases *S1P* and *S2P* that activate AT6 and the CREB proteins by cleavage (Ye et al., 2000; Asada et al., 2011). Included in the group are lesser known transcripts including *IRE1β, ATF6β*, and *NUCB1*.

**Figure 3:**
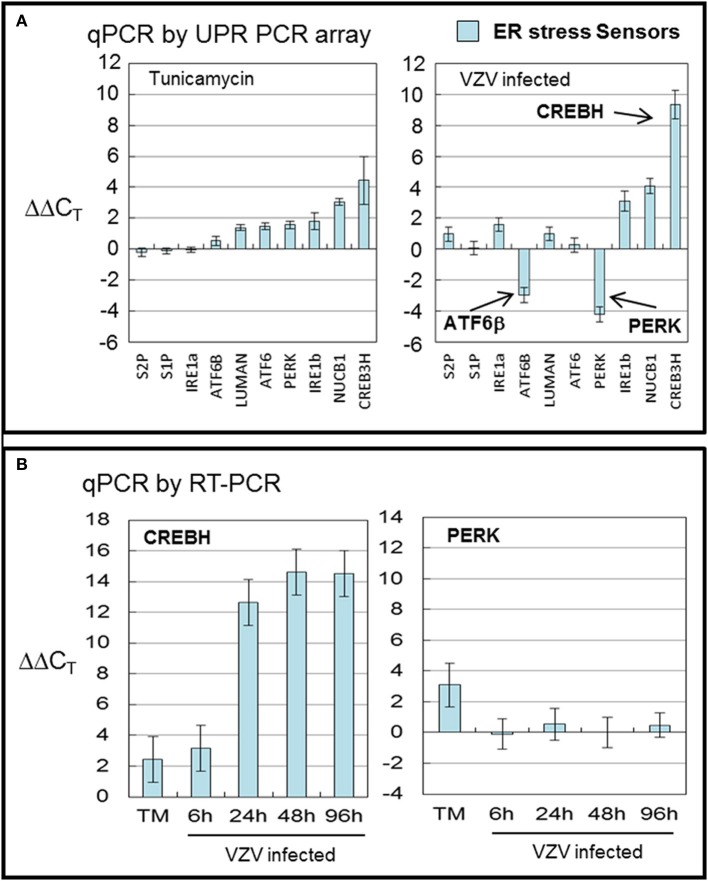
**VZV infection significantly upregulated the transcription factor *CREBH***. Human fibroblast cells (MRC-5) were grown in tissue culture plates then infected with VZV-32 infected MRC-5 cells or treated with tunicamycin (TM), a N-glycosylation inhibitor. At 72 hpi, RNA was processed as described in legend to Figure 2. All gene transcription measurements were graphed for tunicamycin treated and VZV infected cell samples. **(A)** By measurements using the UPR specific PCR array, VZV infected cells showed significant upregulation of *CREBH* with downregulation of *PERK* and *ATF6β*. Tunicamycin treatment upregulated to a lesser extent all stress SENSORs. **(B)** To assess some of the measurements by the UPR array, cDNA from VZV infected and tunicamycin treated cells was submitted for real-time (RT-) PCR using primers specific to *CREBH* and *PERK* (see Methods section for primer information). Error bars correspond to standard deviation when averaging.

CREBH, the cAMP responsive element binding protein H, is an ER anchored transcription factor implicated in nutrient metabolism and the proinflammatory response. VZV infected cells displayed more transcripts of *CREBH* and fewer of *ATF6β* and *PERK* (all with *p* < 0.001) than in TM treated cells (Figure 3A). TM treatment generally upregulated all ER sensor transcripts with *CREBH* the most upregulated. *CREBH* transcription has previously been described as upregulated in hepatocytes and has been associated with lipid synthesis and acute phase transcription in T-cells (Zhang et al., 2006, 2012). More recently, CREBH as a transcription factor has been described as increasing the capacity of the secretory pathway (Barbosa et al., 2013). *ATF6β* and *ATF6α* share similar structures but differ in function. In particular, *ATF6β* has been reported to inhibit transcription of *ATF6α* one of the primary ER stress sensors (Thuerauf et al., 2007).

In order to confirm the results of the UPR specific PCR array, we carried out qPCR measurements using primers to *CREBH* and *PERK* (Figure 3B) in TM treated cells and at several timepoints in VZV infected cells. Those measurements confirm the upregulation of *CREBH* by TM treatment but particularly in VZV infected cells (*p* < 0.01). However, the downregulation of *PERK* in VZV infected cells was not confirmed.

### VZV infected cells exhibited uneven fold gene transcription

Within the FOLD group, the largest, there are 32 wells with primers to eight HSP-70 homologs including *HSPA5/BiP* and *SIL1/BaP*; five DNAJ HSP-40 homologs including *DNAJB9/ERdj4* and *DNAJC10/ERdj5*; twelve wells contain primers to transcripts encoding ER lumen folding components including *CALR* and *CANX*; three components of the folding chaperonin complex and finally four components in the ER membrane including *RPN1* and *SEC63*. Many of these transcripts encode proteins which assist secretory protein folding but also sense misfolded proteins in the ER (Schroder, 2008). For example, DNAJC10/ERdj5 is a disulfide reductase that associates with ERAD component EDEM (Hagiwara et al., 2011).

**Figure 4:**
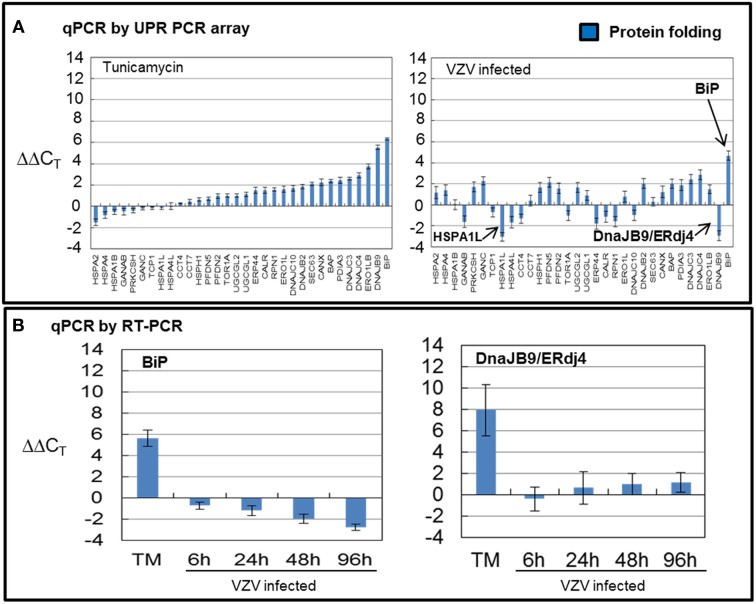
**VZV infected cells exhibited uneven transcription of protein folding genes**. Human fibroblast cells (MRC-5) were grown in tissue culture plates then infected with VZV-32 infected MRC-5 cells or treated with tunicamycin (TM), a N-glycosylation inhibitor. At 72 hpi, RNA was processed as described in legend to Figure 2. All gene transcription measurements were graphed for tunicamycin treated and VZV infected cell samples. **(A)** Using the UPR specific PCR array, tunicamycin treated cells exhibited significant upregulation of transcripts of FOLD chaperones while VZV infected cells exhibited a much more uneven pattern of up or down regulation of FOLD transcripts. In particular, *ERdj4/DNAJB9* and *HSPA1L* were downregulated with only *BiP* showing upregulation. **(B)** To assess some of the measurements by the UPR array, cDNA from VZV infected and tunicamycin treated cells was submitted for real-time (RT-) PCR, using primers specific to *BiP* and *ERdj4/DNAJB9* (see Methods section for primer information). Error bars correspond to standard deviation when averaging.

VZV infected cells exhibited very uneven transcription of folding chaperones (FOLD) while TM treatment robustly upregulated transcription of these chaperones particularly *BiP* (Figure 4A). Measurements with the UPR specific PCR array showed VZV infected cells upregulated *BiP* while downregulating *DNAJB9/ERdj4* and *HSPA1L*. In order to reassess these observations, we carried out qPCR measurements of *BiP* and *DNAJB9/ERDj4* using primers specific to those transcripts (Figure 4B) and found that neither the upregulation of *BiP* nor downregulation of *DNAJB9/ERDj4* was confirmed. Rather the qPCR results found *BiP* to be moderately downregulated as the infection progressed to more cells (*p* < 0.05). However, we also reassessed the regulation of the ER-co-chaperone DNAJC10/ERdj5 and found a correlation with the UPR-specific array (data not shown).

### VZV infected cells significantly downregulated ERAD gene transcription

There are 21 ERAD associated wells that amplify a number of known transcripts that code for proteins that are involved in the degradation of misfolded proteins in the ER through a number of steps: recognition of misfolding (*OS9, PPIA* and *SELS* along with a number of FOLD transcripts), trimming of mannose residues prior to recognition by E3 ubiquitin ligases (*EDEM1* and *EDEM3*), recognition of misfolded proteins by E3 ubiquition ligases (*DERL3/HRD1, DERL2, DERL1, HERP, RNF5*, and associated factors *SEL1L* and *FBX06*), exportation to the cytosolic side of the ER membrane (*SEC62*) where the VCP/p97 complex poly-ubiquitinates protein substrates before extracting/clipping the protein from the membrane to be ultimately degraded in the cytosol by the proteasome (Schroder, 2008; Merulla et al., 2013). The *VCP/p97* complex includes its cofactors *UFD1L* and *NPLOC4* and regulators *ATAXIN3* and *ARMET/erasin* as well as the E2 ubiquitin-conjugators *UBE2J2* and *UBE2G2* (Ballar et al., 2011). Finally, there is a cytosolic protease, *USP14*, included in this grouping.

**Figure 5:**
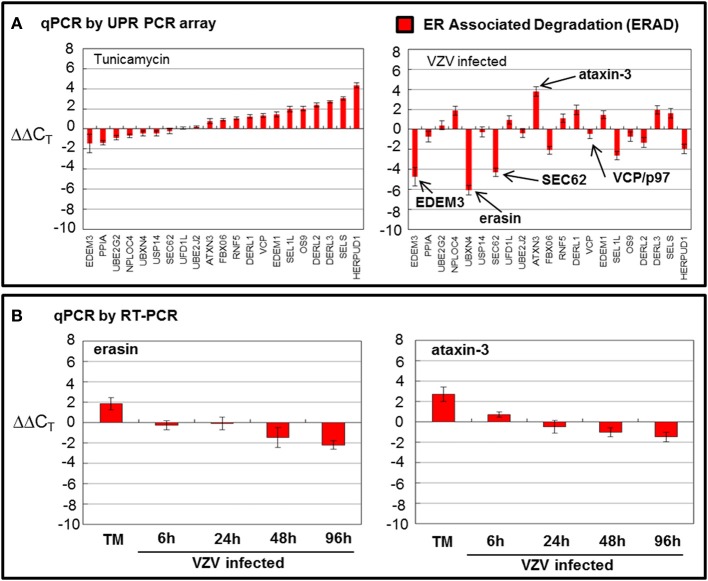
**VZV infection moderately downregulated ER associated degradation genes**. Human fibroblast cells (MRC-5) were grown in tissue culture plates then infected with VZV-32 infected MRC-5 cells or treated with tunicamycin (TM), a N-glycosylation inhibitor. At 72 hpi, RNA was processed as described in the legend to Figure 2. All gene transcription measurements were graphed for tunicamycin treated and VZV infected cell samples. **(A)** By measurements using the UPR specific PCR array, VZV infected cells showed significant downregulation of several elements of the ERAD pathway: *EDEM3, ERASIN*, and *SEC62*. Tunicamycin treatment, in contrast, upregulated most of the ERAD transcripts. **(B)** In order to assess two of the ERAD transcript measurements by the UPR specific PCR array, RT-PCR was carried out on cDNA from uninfected, tunicamycin treated and VZV infected cells, using primers to *UBXN4/erasin* and *ataxin-3.*

While TM treatment showed almost complete upregulation of ERAD transcripts particularly *HERP* (Figure 5A), VZV infection showed considerable downregulation of ERAD transcript (Figure 5A) where *EDEM3, UBXN4/erasin* and *SEC62* were downregulated. However, *ATAXIN3* was upregulated in VZV infected cells. Both erasin and ATAXIN3 are regulators, positive and negative, respectively, of VCP/p97 (Lim et al., 2009; Liu and Ye, 2012). As noted above, VCP/p97 forms the protein complex in the ER membrane on the cytosolic side that poly-ubiqininates ERAD substrates that are then released into the cytosol to be degraded by the proteasome (Ballar et al., 2011). Downregulation of VCP/p97 via its regulators appeared to reduce ERAD in VZV infected cells. All observed differences were significant with *p* < 0.001. Again, in order to reassess two of the more striking observations from the UPR specific PCR array, we carried out qPCR measurements using primers to *UBXN4/erasin* and *ATAXIN-3* (Figure 5B). These measurements showed *UBXN4/erasin* to be modestly downregulated in VZV infected cells while ATAXIN-3 was essentially unchanged.

### VZV infected cells upregulated transcription of cholesterol synthesis regulator INSIG

**Figure 6:**
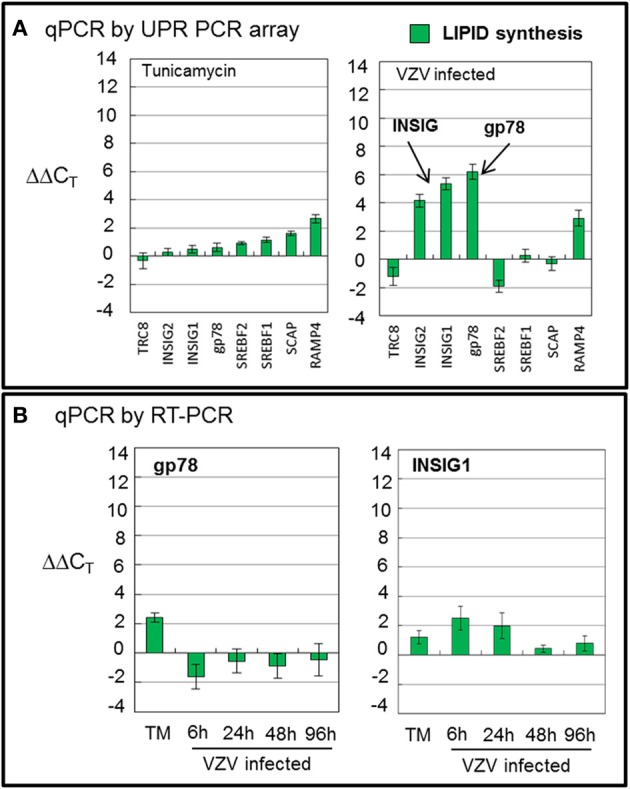
**VZV infection significantly upregulated the cholesterol synthesis associated transcript *INSIG***. Human fibroblast cells (MRC-5) were grown in tissue culture plates then infected with VZV-32 infected MRC-5 cells or treated with tunicamycin (TM), a N-glycosylation inhibitor. At 72 hpi, RNA was processed as described in the legend to Figure 2. **(A)** Transcripts associated with lipid synthesis and metabolism where both VZV infected cells and tunicamycin cells showed similar transcription of *RAMP4;* VZV infected cells in particular showed more transcription of cholesterol synthesis regulator *INSIG*. **(B)** In order to assess two of the lipid transcript measurements by the UPR specific PCR array, RT-PCR was carried out on cDNA from uninfected, tunicamycin treated and VZV infected cells using primers to *AMFR/gp78* and *INSIG1.*

Transcripts associated with lipid synthesis and metabolism such as *RAMP4* showed similar transcription in both VZV infected and TM treated cells but VZV infected cells, in particular, showed increased transcription of cholesterol synthesis regulators *AMFR/gp78* and *INSIG* (Figure 6A). *AMFR/gp78* is an E3 ubiquitin ligase and INSIG is an insulin signaling factor (Flury et al., 2005; Chen et al., 2012). Both are localized to the ER membrane and when activated function together to degrade HMG COA reductase, a cholesterol synthesis enzyme (Jo et al., 2011; Tsai et al., 2012).

In order to reassess the upregulation of *AMFR/gp78* and *INSIG*, we carried out qPCR measurements using primers to each transcript (Figure 6B). Of note, *INSIG* was upregulated in VZV infected cells at early timepoints in agreement with the UPR specific array, while *AMFR/gp78* was not increased. Of note, greater transcription of *INSIG* may lead to reduced cholesterol synthesis in VZV infected cells with the consequence of a more fluid ER in those cells. Cholesterol acts as a stabilizing agent in lipid membranes by supporting adjacent lipid head groups and reducing disorder of the lipid hydrocarbon chains internal to the bilayer (Mouritsen and Zuckermann, 2004). All observed differences were significant with *p* < 0.001.

### VZV infected cells downregulated the transcription factor *C/EBPb* and displayed differential transcription of apoptotic transcripts

**Figure 7:**
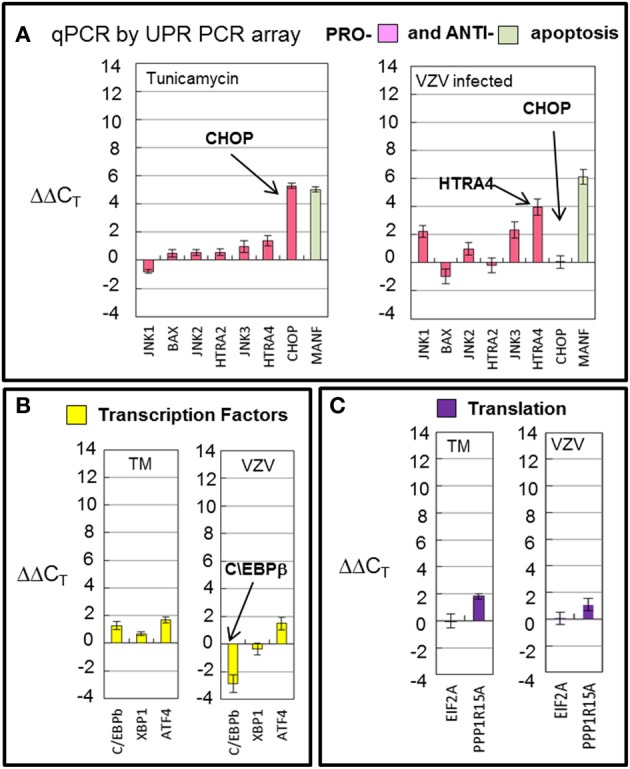
**VZV infection downregulated the transcription factor *C/EBPb* and displayed differential transcription of apoptotic transcripts**. Human fibroblast cells (MRC-5) were grown in tissue culture plates then infected with VZV-32 infected MRC-5 cells or treated with tunicamycin (TM), a N-glycosylation inhibitor. At 72 hpi, RNA was processed as described in the legend to Figure 2. **(A)** Transcription of apoptotic genes differed considerately between VZV infected cells and tunicamycin treated cells. VZV infected cell transcripts showed very fewer *CHOP* transcripts when compared to TM treatment; infected cells had more transcripts associated with cellular apoptosis such as *HTRA4* and MAP kinases *JNK1* and *JNK3*. **(B)** Transcription of cellular transcription factor *C/EBPβ* was significantly downregulated in VZV infected cells as compared to the value in TM treated cells **(C)**. There was no difference between VZV vs. TM treatment for two protein translation associated transcripts. Error bars correspond to standard deviation when averaging.

Transcription of cellular transcription factor *C/EBPβ* was significantly downregulated (*p* < 0.001) in VZV infected cells as compared to the value in TM treated cells (Figure 7A) C/EBPβ is a transcription factor with a large effect on cellular proliferation (Tang and Lane, 2000). Downregulation of this factor may put VZV infected cells into a non-proliferative state. Transcription of apoptotic genes differed considerably between VZV infected cells and TM treated cells. TM treated cells exhibited much higher *CHOP* transcription than VZV infected cells while VZV infected cells showed a greater number of transcripts associated with cellular apoptosis such as *HTRA4* and the MAP kinases *JNK1* and *JNK3* (Figure 7B). Finally there was no difference between the levels of two protein translation associated transcripts in VZV infected cells vs. TM treated cells (Figure 7C).

### Transfection of VZV gE upregulated transcription of *CREBH* and *BiP* while transfection of VZV IE62 did not

**Figure 8:**
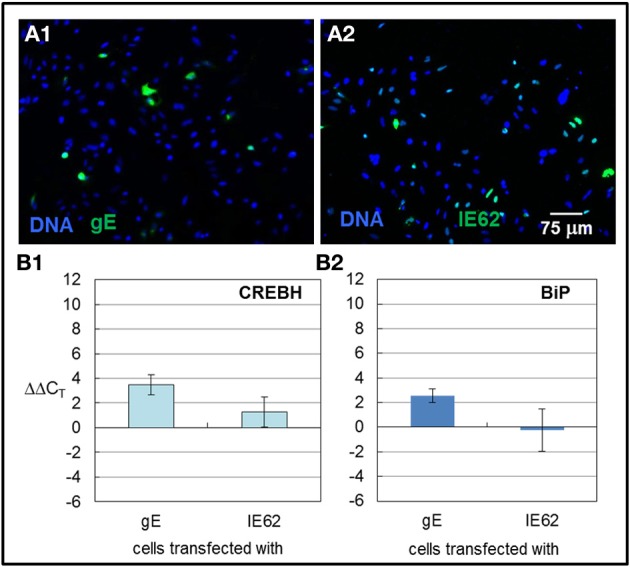
**CREBH and BiP transcription were upregulated in cells transfected with VZV gE but not VZV IE62**. HeLa cells were grown in six well culture plates with or without glass coverslips and subsequently transfected with plasmids encoding the VZV gE glycoprotein or a non-glycosylated VZV IE62 protein, using either Lipofectamine 2000 or ExtremeGene HP transfection reagents. 6 h after the transfection reagent and plasmid were applied to the cells, the medium was replaced with fresh medium. Some monolayers were processed for microscopy while others were harvested for RNA extraction. The extracted RNA was converted to cDNA and RT-PCR was performed using primers against *CREBH* and *BiP*. **(A1,A2)** Representative images of cells transfected with VZV gE **(A1)** or VZV IE62 **(A2)**. (**B1,B2)** RT-PCR values were normalized to *GAPDH* and then differences to values measured for cells that were only treated with transfection reagent alone were computed.

In 2011, we found that transfecting cells with VZV glycoprotein genes led to increased autophagosome production and inflation of the ER. Transfection with VZV IE62 led to neither increased autophagosomes nor a larger ER. Therefore, we measured by qPCR whether CREBH and BiP transcription was increased by transfection of a glycoprotein vs. a non-glycoprotein that is also the major transactivator encoded by VZV. Transfection with a plasmid encoding VZV gE under the CMV immediate early promoter led to approximately 10% of transfected cells (Figure 8A1) while transfection with VZV IE62 also under the CMV immediate early promoter led to a larger number, approximately 40%, of transfected cells (Figure 8A2). Even though a low fraction of cells were transfected with VZV gE, increased transcription of *CREBH* and *BiP* were observed in those cells (Figures 8B1,B2) whereas not in cells transfected with VZV IE62 even though many more cells were transfected in those samples (*p* < 0.01). We therefore conclude that expression of a single VZV glycoprotein gene in cells is sufficient to activate the CREBH arm of the UPR.

## Discussion

We have previously documented that VZV infection induces an autophagic response in infected cells. The basic observation of this report is that VZV infected cells differentially activate the UPR to ER stress as compared to tunicamycin treated cells. The most straightforward explanation for this observation is that tunicamycin treatment produces many misfolded glycoproteins while VZV infection produces an overabundance of normally folded glycoproteins. The elements of the UPR activated in each situation would likely differ. As compared to the positive control of tunicamycin treated cells, VZV infected cells showed increased transcription of a gene associated with decreased cholesterol synthesis as well as increased transcription of the ER stress sensor and transcription factor, *CREBH*. At the same time, VZV infected cells showed decreased transcription of genes associated with ERAD and apoptosis. We hypothesize that this transcriptional profile is compatible with the infected cell attempting to accommodate the influx of viral glycoproteins by greatly increasing the capacity of the ER. For example, increased transcription of *AMFR* (*gp78*) and *INSIG* is associated with a decrease in cholesterol synthesis via degradation of HMG-CoA reductase, an enzyme necessary for cholesterol synthesis (Jo et al., 2011; Tsai et al., 2012). Decreased cholesterol content would increase the lability of the ER membrane and facilitate expansion of the ER (Mouritsen and Zuckermann, 2004).

We also found that some differences in transcription between tunicamycin treated cells vs. VZV infected cells as measured by the UPR specific PCR array could not be confirmed by qPCR, using individual primers selected by our laboratory. The reason behind these discrepancies is unclear but may center around two possibilities: (i) The choice of primers in the PCR array vs. those used in the qPCR measurements or (ii) the asynchronous nature of VZV infection. In general, there was better agreement between tunicamycin treated values as measured by the UPR-specific PCR array and individual qPCR assays. The biggest differences were observed in values measured from VZV infected cells. The latter scenario suggests that asynchronous VZV infection may play a role; for example, the input virus is always extremely low, such that some cells within a monolayer will remain uninfected even at 72 hpi. This same scenario may explain why we have observed similar unexpected differences in experiments to measure protein expression, for example, BiP. Because of increased VZV-induced autophagy, we predicted increased BiP production in infected cells. However, we have observed variable changes in BiP protein expression following VZV infection. Nevertheless, the main conclusions of this report were confirmed by both assays, namely, the significant upregulation of CREBH as well as the more modest upregulation of the cholesterol regulator INSIG.

A key question is whether the infected cell is responding to abundant viral glycoprotein expression through normal mechanisms or alternatively, does the virus encode its own proteins which manipulate the UPR. Recently, Burnett et al found that HSV ICP0 transactivated elements of the UPR and in turn was transactivated by the UPR itself via an ERSE promoter element in the HSV ICP0 gene (Burnett et al., 2012). Similarly, both the human and murine strains of the beta herpesvirus cytomegalovirus (CMV) encode proteins that manipulate the UPR—proteins that the VZV genome does not encode (Isler et al., 2005; Xuan et al., 2009; Qian et al., 2012; Stahl et al., 2013). VZV does encode a homolog of HSV ICP0—ORF61, but its promoter region does not appear to have any of the known UPR promoter elements: ERSE, ERSE-II, and UPRE based on bioinformatics searches (data not shown). It would be interesting to test VZV ORF61 against a luciferase reporter construct containing the BiP promoter element in future experiments.

In further support of our hypothesis that abundant expression of VZV glycoproteins contributes to the activation of the UPR in a specific way that leads to an enlarged ER and increased autophagosome production, we found that transfection of the VZV gE gene led to increased *CREBH* and *BiP* transcription. We observed abundant VZV gE protein in the ER/Golgi after transfection. In contrast, transfection with VZV IE62, a non-glycosylated viral protein, did not lead to increased transcription of either transcript. Obviously, the IE62 protein never enters the ER/Golgi. These results confirm and expand our 2011 report that transfection with VZV glycoprotein genes resulted in increased ER size and increased autophagosome production. The UPR is known to upregulate autophagy (Yorimitsu et al., 2006).

In summary, even though both tunicamycin treatment and VZV infection induced an UPR, the profiles of UPR related genes were different after the two analyses. The UPR in VZV infected cells exhibited greatly increased *CREBH* and cholesterol synthesis regulation transcription and diminished ERAD transcription. The transcription patterns appeared to correlate with increasing ER capacity secondary to increasing viral glycoprotein synthesis in the infected cell. Of importance, the CREBH data were totally unexpected, based on all prior VZV research, and would never have been uncovered in the absence of the UPR array data described in this report.

### Conflict of interest statement

The authors declare that the research was conducted in the absence of any commercial or financial relationships that could be construed as a potential conflict of interest.
